# Characterization of *Staphylococcus aureus* isolates from raw milk sources in Victoria, Australia

**DOI:** 10.1186/s12866-016-0789-1

**Published:** 2016-07-29

**Authors:** Kate McMillan, Sean C. Moore, Catherine M. McAuley, Narelle Fegan, Edward M. Fox

**Affiliations:** 1 CSIRO Agriculture and Food, PO Box 745, Archerfield BC, QLD 4108 Australia; 2 CSIRO Agriculture and Food, 671 Sneydes Road, Werribee, VIC 3030 Australia

**Keywords:** Staphylococcus aureus, Dairy farm, PFGE, MLST, Antibiotic sensitivity, Staphylococcal enterotoxin, Milk

## Abstract

**Background:**

Highly pathogenic strains of *Staphylococcus aureus* can cause disease in both humans and animals. In animal species, including ruminants, *S. aureus* may cause severe or sub-clinical mastitis. Dairy animals with mastitis frequently shed *S. aureus* into the milk supply which can lead to food poisoning in humans. The aim of this study was to use genotypic and immunological methods to characterize *S. aureus* isolates from milk-related samples collected from 7 dairy farms across Victoria.

**Results:**

A total of 30 *S. aureus* isolates were collected from milk and milk filter samples from 3 bovine, 3 caprine and 1 ovine dairy farms across Victoria, Australia. Pulsed Field Gel Electrophoresis (PFGE) identified 11 distinct pulsotypes among isolates; all caprine and ovine isolates shared greater than 80 % similarity regardless of source. Conversely, bovine isolates showed higher diversity. Multi-Locus Sequence Typing (MLST) identified 5 different sequence types (STs) among bovine isolates, associated with human or ruminant lineages. All caprine and ovine isolates were ST133, or a single allele variant of ST133. Two new novel STs were identified among isolates in this study (ST3183 and ST3184). With the exception of these 2 new STs, eBURST analysis predicted all other STs to be founding members of their associated clonal complexes (CCs). Analysis of genetic markers revealed a diverse range of classical staphylococcal enterotoxins (SE) among isolates, with 11 different SEs identified among bovine isolates, compared with just 2 among caprine and ovine isolates. None of the isolates contained *mecA*, or were resistant to oxacillin. The only antibiotic resistance identified was that of a single isolate resistant to penicillin; this isolate also contained the penicillin resistance gene *blaZ*. Production of SE was observed at 16 °C and/or 37 °C in milk, however no SE production was detected at 12 °C.

**Conclusion:**

Although this study characterized a limited number of isolates, bovine-associated isolates showed higher genetic diversity than their caprine or ovine counterparts. This was also reflected in a more diverse SE repertoire among bovine isolates. Very little antibiotic resistance was identified among isolates in this study. These results suggest maintaining the milk cold chain will minimise any risk from SE production and highlights the need to prevent temperature abuse.

## Background

*Staphylococcus aureus* is a Gram-positive bacterium that often resides harmlessly in a wide range of niches from environmental samples to the skin and mucosa of humans and other animals. However some strains are highly pathogenic and are frequently implicated as the causative agent of both human and animal disease. In humans *S. aureus* is a major contributor to food poisoning, pneumonia and post-operative wounds [1]. In animal species, including ruminants, *S. aureus* is capable of causing severe cases of mastitis, arthritis and urinary tract infections, and can also cause sub-clinical mastitis [2].

Mastitis is an inflammatory infection of the mammary glands caused by a range of bacteria but frequently due to *S. aureus* infection [3]. Mastitis has a detrimental effect on the health of the animal and the quality and quantity of milk it’s able to produce. Mastitis is considered one of the most costly dairy farm diseases worldwide [4]. In Australia the financial impact of *S. aureus* on dairy production is estimated to cost up to $150 million per annum [5]. Milk produced from cows infected with mastitis also poses a threat to human health. Cows infected with mastitis routinely shed large numbers of bacteria into their associated milk supply. Contaminated milk potentially contains pathogenic strains that are capable of causing food poisoning [6].

*S. aureus*-associated food poisoning in humans and similarly intramammary infection in animals is produced by strains with the capacity to produce a variety of virulence factors [7]. These virulence factors include the production of antigens, toxins and various resistance proteins. The main virulence factor attributed to staphylococcal food poisoning is a group of highly heat resistant super antigens called staphylococcal enterotoxins (SE), which can survive pasteurisation [8]. There are over 20 described SEs to date, with the five main serological groups of significance to foodborne illness being SEA, SEB, SEC, SED and SEE, all of which have been identified in outbreaks traced to the consumption of raw milk [6, 9]. Many mastitis-associated strains of *S. aureus* also produce enterotoxins as well as other factors including staphylococcal toxic shock syndrome toxin and exfoliative toxins [10]. The role of virulence factors in disease causation is not as definitive as foodborne illness; with a plethora of factors likely to be involved in disease pathogenesis [11]. In human and animal disease alike, pathogenicity can be aided by the bacteria’s ability to resist treatment by antibiotics. Antibiotic resistance may be conferred by a number of genetic markers including the *mecA* gene, encoding for the production of a penicillin binding protein called PBP2a, which can inhibit the effectiveness of a range of antibiotics [12]. More recently *mecC*-mediated resistance has also emerged among *S. aureus* isolates [13].

On farms, these pathogenic strains can originate from a variety of sources including the animal itself, the skin of farm staff, the environment, and other animals visiting the farm [14]. These strains can easily transmit from one source to another via different routes of transmission including, animal to animal contact, farm bedding, milking equipment and the hands of milkers [15]. To gain insight into the relatedness of strains, potential sources of infection, routes of transmission and presence of virulence and resistance markers, genotypic and phenotypic methods can be employed. A combination of genotypic methods offers the greatest comparisons over a standalone method. Pulsed field gel electrophoresis (PFGE), which is based on the migration of macro-restriction fragments is frequently termed the gold standard approach. PFGE enables epidemiological comparisons to be drawn whereas multi locus sequence typing (MLST), which is based on the sequence polymorphism of seven conserved housekeeping genes, provides greater evolutionary comparisons [16]. MLST also offers the advantage that it is highly reproducible making it an excellent tool for global comparisons of population structures [17]. Genome sequencing can be utilised to identify the presence of important genetic markers such as those associated with virulence or antibiotic resistance. It can also determine their potential spread to other bacterial species, by identifying if these genes are located on mobile genetic elements [18]. Phenotypic methods can then be used to provide information on the antibiotic sensitivity, and level of toxin production of the isolates [19, 20].

The sale of raw goats’ milk is permitted in four Australian states: New South Wales, South Australia, Western Australia and Queensland. Although the sale of raw cow and sheep milk is currently illegal in Australia, there could be a push for raw milk to be legalised in the future if its popularity continues to soar [21]. The aim of this study was to characterize *S. aureus* isolated from bovine, caprine, and ovine dairy farms in Victoria, by studying the genetic diversity of the isolates, gain knowledge on the presence of virulence markers and their significance in contaminated milk, and get an understanding of the level of antimicrobial resistance in an Australian context as there is limited data available in this area. The importance of this study is highlighted by the recent increase in community acquired *S. aureus* infection and an ever increasing demand for raw milk sales overseas [22].

## Results

### PFGE subtyping

**Fig. 1:**
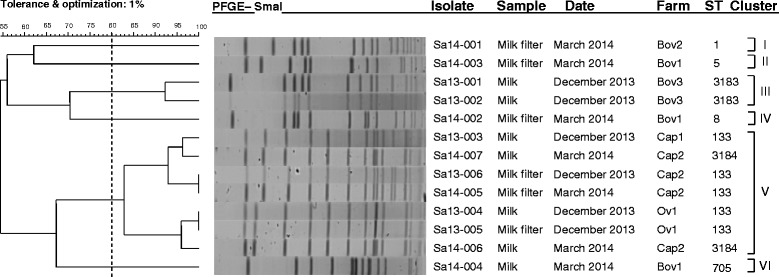
Dendrogram depicting the PFGE profiles of the 13 isolates collected from the Victorian dairy farms. The corresponding farm, sample type, isolation date, animal class and ST are shown

PFGE of the *SmaI* macro-restriction digests yielded 13 isolates among samples collected, with 11 unique banding patterns containing 10–18 fragments (if multiple isolates from the same sample shared a PFGE profile, only 1 of these was included in further analyses). This comprised 3 pulsotypes among the 8 isolates from Bov1 farm samples; 1 pulsotype among the 4 Bov2 isolates; 1 pulsotype among the 4 Bov3 isolates; 1 pulsotype among the 4 Cap1 isolates; 4 pulsotypes among the 5 Cap2 isolates; and 2 pulsotypes among the 5 Ov1 isolates. Using a similarity coefficient cut off point of 80 % as previously described [23], 6 clusters (I, II, III, IV, V and VI) were defined (Fig. 1). Isolates obtained within farms all belonged to the same cluster, except for the 3 strains collected from a single bovine farm which all belonged to their own individual clusters (II, IV and VI). An association with animal type was observed for these clusters; cluster I, II, III, IV and VI consisted entirely of bovine isolates and cluster V entirely of ovine and caprine isolates. An association with sequence type was also observed for cluster V with 5 of the 7 isolates being ST133 (the other 2 isolates were ST3183, a single allele variant of ST133); similarly cluster III comprised ST3183 isolates.

PFGE subtypes displaying indistinguishable banding patterns were only observed within farms. A PFGE pulsotype shared by 2 isolates on the ovine farm (Sa13-004 and Sa13-005) indicated the same pulsotype of *S. aureus* was present in the milk filter and its associated milk sample. Indistinguishable pulsotypes were identified on caprine farm Cap2 (Sa13-006 and Sa14-005) in samples taken 3 months apart, suggesting persistence of a strain on the farm from summer through to autumn the following year. This persistent strain identified on the milk filters closely resembled 2 milk isolates collected in summer from the same farm (Sa14-007) and another caprine farm (Sa13-003), respectively.

### MLST subtyping of isolates

**Fig. 2:**
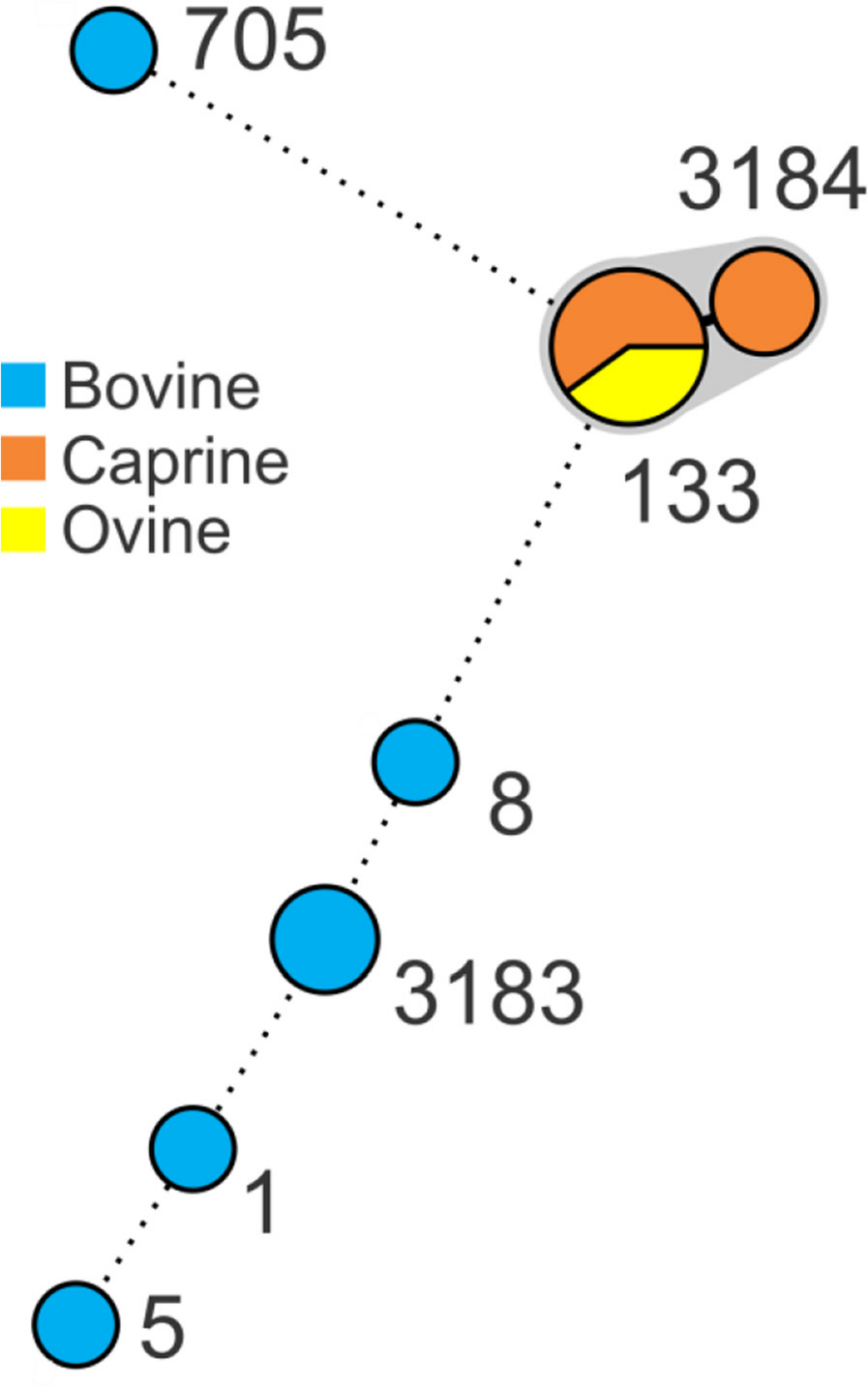
Minimum Spanning Tree based on the MLST data for each isolate. Numbers indicate ST of each node. Isolates differing by only a single allele are partitioned (*grey shading*). Novel STs identified in this study were ST3183 and ST3184

Analysis of the MLST data indicated a total of 7 STs among the isolates, including 2 novel STs (ST3183 and ST3184; Fig. 2). Isolates derived from individual farms all had the same ST except for the farm bov1 which had isolates belonging to ST5, ST8 and ST705, and Cap 2 which had isolates belonging to ST133 and ST3184. All ovine and caprine isolates were ST133 with the exception of 2 caprine isolates (ST3184) which were a single locus variant of ST133 at the *arcC* locus. Bovine isolates grouped into ST1, ST5, ST8, ST705 and a novel ST, ST3183 (a single locus variant of ST97 in the *tpi* locus). All ovine and caprine isolates populated CC133 whereas the bovine isolates were assigned to CC1, CC5, CC8, CC97 or CC705.

A search of the online MLST database [http://saureus.mlst.net/; 20] returned 216 isolates from Australia which primarily originated from remote communities in Western Australian, none of which were of dairy milk origin. The predominant STs represented among Australian isolates were ST1, ST5, ST6, ST8 and ST12, 3 of which were also identified in this study (ST1, ST5 and ST8). A global search returned 558 *S. aureus* isolates of dairy milk origin of which 472 were from bovine milk, 18 were from ovine milk and 6 from caprine milk. Sixty-two isolates could not be assigned an animal type, due to insufficient records; these 62 isolates were excluded from the analysis. The database was then split into animal type (ovine, caprine and bovine) as *S. aureus* lineages are considered host specific [14]. The eBURST software [24] was then used to construct a population scatter diagram to compare the isolates of this study with the global dataset to understand the global population structure.

**Fig. 3:**
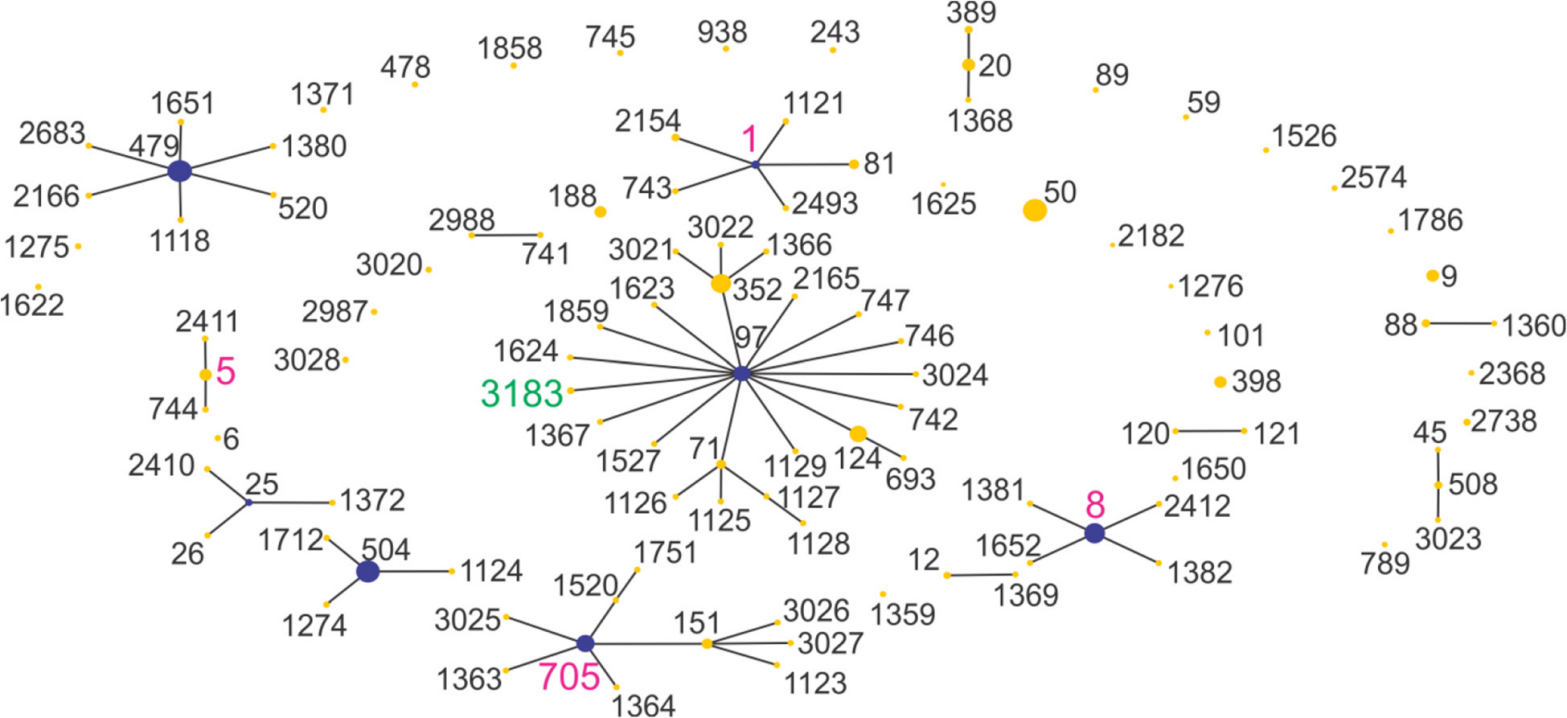
Population snapshot of all bovine isolates. *Pink labels* represent isolate STs from this study also in the eBURST database, whereas the *green label* (ST3183) is unique to this study. The putative founding ST is coloured blue, where this could be predicted

**Fig. 4:**
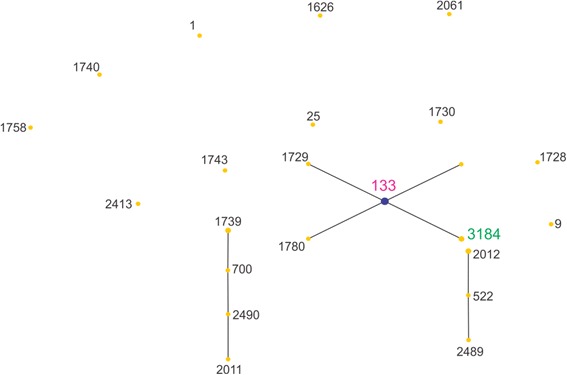
Population snapshot of all ovine and caprine isolates. *Pink labels* are isolate STs from this study also in the eBURST database, and the *green label* isolate ST (ST3184) was identified in this study but not in the eBURST database. The putative founding ST is *coloured blue*, where this could be predicted

A population scatter diagram of the bovine milk showed 6 main primary groups (Fig. 3). STs from this study fell into the primary founder clusters, indicating relatedness to global ancestral lineages (Table 1). The eBURST analysis of caprine and ovine groups was combined into the class of small ruminants due to small sample size and high correlation in STs seen, and showed a dominance of ST133 (Fig. 4 and Table 2). Of the previously identified STs also identified in this study, interrogation of the *S. aureus* online MLST database showed all ST133 and ST705 isolates were of ruminant origin whereas ST1, ST5, ST8 and ST97 contained isolates of various origins.

**Table 1:** eBURST Groups of bovine dairy isolates in MLST database, including number of isolates, number of STs per eBURST group and the likely predicted founder (6 out of 7 matching loci)

Group^a^	No. of isolates	No. of STs	Predicted founder
1	117	23	97
2	43	10	705
3	49	7	479
4	17	6	1
5	38	5	8
6	50	4	504
7	7	4	25
8	16	3	20
9	5	3	508
10	11	3	5
11	3	2	None
12	2	2	None
13	5	2	None
14	5	2	None

^a^Data taken 16th February 2015 (Table does not include 30 singletons)

**Table 2:** eBURST Groups of ovine dairy isolates in MLST database, including number of isolates, number of STs per eBURST group and the likely predicted founder (6 out of 7 matching loci)

Group^a^	No. of isolates	No. of STs	Predicted founder
1	13	5	133
2	5	4	Multiple candidates
3	4	3	522

^a^Data taken 16th February 2015 (Table does not include 11 singletons)

### In-silico analysis of virulence gene markers

All isolates, regardless of animal type or farm contained the virulence gene *nuc* (Table 3). The greatest variation in staphylococcal enterotoxin gene prevalence was seen in bovine isolates. The *seg, sei, sem*, *sen, seo, yent1* and *yent2* genes were present in two isolates. Other SE genes identified in bovine isolates included *sec, sed, seh, sej*, *sel,* and *ser*. Both ovine and 3 caprine isolates contained *sec* and *sel*. Five isolates harbored the *tst* gene which codes for toxic shock syndrome toxin (TSST-1): 1 bovine, both ovine, and 2 caprine isolates.

**Table 3:** Presence of key virulence genes among isolates in this study

			Enterotoxin genes																	Pathogenesis genes			
	Isolate	Farm	*sea*	*seb*	*sec*	*sed*	*see*	*seg*	*seh*	*sei*	*sej*	*sek*	*sel*	*sem*	*sen*	*seo*	*sep*	*seq*	*ser*	*yent1*	*yent2*	TSST-1	*nuc*
Bovine	Sa14-002	Bov1	-^a^	-	-	-	-	-	-	-	-	-	-	-	-	-	-	-	-	-	-	-	+^b^
	Sa14-003	Bov1	-	-	-	+	-	+	-	+	+	-	-	+	+	+	-	-	+	+	+	-	+
	Sa14-004	Bov1	-	-	+	-	-	+	-	+	-	-	+	+	+	+	-	-	-	+	+	+	+
	Sa14-001	Bov2	-	-	-	-	-	-	+	-	-	-	-	-	-	-	-	-	-	-	-	-	+
	Sa13-001	Bov3	-	-	-	-	-	-	-	-	-	-	-	-	-	-	-	-	-	-	-	-	+
	Sa13-002	Bov3	-	-	-	-	-	-	-	-	-	-	-	-	-	-	-	-	-	-	-	-	+
Caprine	Sa13-003	Cap1	-	-	+	-	-	-	-	-	-	-	+	-	-	-	-	-	-	-	-	-	+
	Sa13-006	Cap2	-	-	-	-	-	-	-	-	-	-	-	-	-	-	-	-	-	-	-	-	+
	Sa14-005	Cap2	-	-	-	-	-	-	-	-	-	-	-	-	-	-	-	-	-	-	-	-	+
	Sa14-006	Cap2	-	-	+	-	-	-	-	-	-	-	+	-	-	-	-	-	-	-	-	+	+
	Sa14-007	Cap2	-	-	+	-	-	-	-	-	-	-	+	-	-	-	-	-	-	-	-	+	+
Ovine	Sa13-004	Ov1	-	-	+	-	-	-	-	-	-	-	+	-	-	-	-	-	-	-	-	+	+
	Sa13-005	Ov1	-	-	+	-	-	-	-	-	-	-	+	-	-	-	-	-	-	-	-	+	+

^a^-, negative

^b^+, positive

### Antibiotic sensitivity testing

Based on CLSI breakpoints, of the 13 isolates only a single isolate from farm bov1 milk filter displayed resistance towards penicillin. This was also the only isolate to carry the penicillin-resistance gene marker *blaZ*. All other isolates were susceptible to penicillin, with the exception of the ovine isolate Sa13-005, which showed intermediate resistance. It should be noted that based on EUCAST guidelines, this isolate would be considered sensitive to penicillin. All isolates were sensitive to all other antibiotics tested (Table 4). No resistance to oxacillin was identified and no isolates harboured *mecA* or *mecC*, indicating all isolate were methicillin sensitive. No resistance to erythromycin, vancomycin and tetracycline was observed, which correlated with the absence of *ermA*, *ermC*, *vanA*, *vanB*, *vanC*, *tetK* or *tetM* resistance genes in all isolates. The *aacA*-*aphD* gentamicin, tobramycin and kanamycin resistance determinant was similarly absent in all isolates.

**Table 4:** Antibiotic sensitivity of isolates in this study

Antibiotic	Class	Sensitive	Intermediate	Resistant
Ceftriaxone^a^	Cephems	100 % (*n =* 13)	0 % (*n =* 0)	0 % (*n =* 0)
Ciprofloxacin	Fluoroquinolones	100 % (*n =* 13)	0 % (*n =* 0)	0 % (*n =* 0)
Erthromycin	Macrolides	100 % (*n =* 13)	0 % (*n =* 0)	0 % (*n =* 0)
Oxacillin	Penicillins	100 % (*n =* 13)	0 % (*n =* 0)	0 % (*n =* 0)
Penicillin	Penicillins	84.6 % (*n =* 11)	7.7 % (*n =* 1)	7.7 % (*n =* 1)
Tetracycline	Tetracyclines	100 % (*n =* 13)	0 % (*n =* 0)	0 % (*n =* 0)
Vancomycin	Glycopeptides	100 % (*n =* 13)	0 % (*n =* 0)	0 % (*n =* 0)

^a^Breakpoints based on FDA guidelines [57]

Brekpoints are based on CLSI guidelines, with the exception of ceftriaxone, which uses the FDA brekpoints

### Toxin production immunoassay

**Fig. 5:**
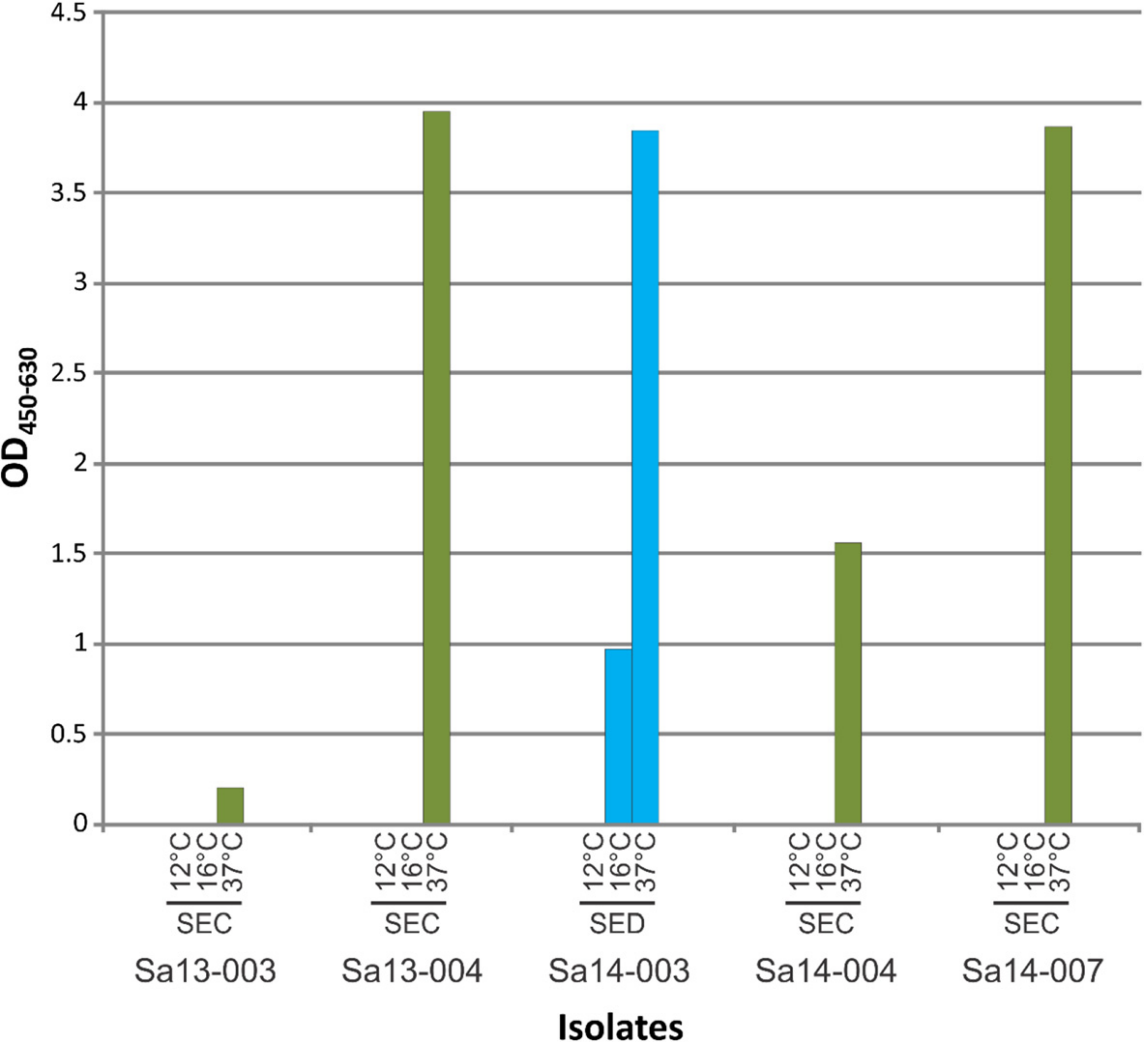
Level of staphylococcal enterotoxin detected in milk at 12 °C for 72 h, 16 °C for 48 h, or at 37 °C for 18 h. Five isolates, each differing by pulsotype, were screened. *Green*, SEC; *blue*, SED

Detection of toxin expression of 5 isolates was carried out for the 5 main staphylococcal enterotoxins (SEA*,* SEB*,* SEC*,* SED and SEE) at 12 °C for 72 h, 16 °C for 48 h and 37 °C for 18 h; both 12 °C and 16 °C were selected to reflect the impact of temperature abuse and 37 °C to represent human body temperature. Isolates grown at 12 °C did not show significant growth (<0.38 log CFU/ml), whereas those grown at both 16 °C and 37 °C reached late exponential or early stationary phase. At 12 °C for 72 h no toxin was detected, however at 16 °C for 48 h SED production by Sa14-003 was detected. At 37 °C all isolates with *sec* tested produced detectable SEC and Sa14-003 produced a higher amount of SED relative to its production at 16 °C (Fig. 5).

## Discussion

The contamination of dairy herds and raw milk by *S. aureus* remains an important issue in dairy food production. Its public health significance is evidenced by the plethora of foodborne disease outbreaks resulting from contaminated dairy products, some of which are reviewed by Hennekinne et al. [7], including one of the largest foodborne outbreaks attributed to a bacterial contaminant on record involving 13,420 infectees [25]. Aside from this, the cost burden to the dairy community is immense; udder infection of herd animals can lead to significant decreases in milk production, with associated veterinary and treatment costs, and in some cases require culling [7]. This study incorporated genotypic, phenotypic and immunological methods to examine population structure, antimicrobial resistance, carriage of staphylococcal enterotoxin genes and production of the 5 classical enterotoxins (SEA, SEB, SEC, SED, and SEE) of *S. aureus* isolated from dairy farms across Victoria, Australia. Due to its temperate climate and mineral rich pastures Victoria is an important contributor to milk production in Australia producing roughly two thirds of the supply, which itself amounted to 9.2 billion litres in the 2012/13 period [26]. Understanding the genetic diversity of *S. aureus* from dairy cattle can help identify geographically predominant clones as well as animal and herd specific clones which can aid in the implementation of on farm control measures for mastitis.

PFGE analysis of isolates from individual farms indicated a clonal population on each farm, with isolates sharing 83 % or greater similarities; with the exception being bovine farm Bov1, which showed a more diverse population (Fig. 1). Caprine and ovine isolates clustered together, suggesting these isolates show a closer genetic relationship, relative to the other bovine isolates; this is in agreement with previous analysis of ruminant isolates [27]. In contrast to this, the bovine isolates showed a higher genetic diversity relative to each other. Interestingly, the Sa14-004 bovine isolate clustered closer to the caprine and ovine isolates, suggesting it is more distantly related to the other bovine genomes.

MLST provides an excellent tool for investigating the population structure of *S. auerus* globally [28]. Analysis of the isolates in this study identified 5 known STs (ST1, ST5, ST8, ST133 and ST705), in addition to 2 novel STs not previously described: ST3183 and ST3184 (Fig. 2). As was observed with the PFGE analysis, isolates clustered into groups indicating greater variation among bovine isolates, contrasting to the clonal nature of the ovine and caprine isolates. These findings support the hypothesis that *S. aureus* is a clonal organism that spreads from animal to animal and as such the number of strains detected per farm is low [29, 30]. Exceptions may be accounted for by differences in farm management styles on individual farms and variations in some herds.

The eBURST v3 algorithm was used to examine the population structure of isolates in this study relative to global bovine, caprine or ovine isolates. The global population structure of bovine milk-associated *S. aureus* STs formed 14 primary groups (Table 1 and Fig. 3). With the exception of ST3184, bovine isolates from this study were representative of the predicted founding ST of their associated group or CC. Two of the bovine isolates from this study fell into CC97 and 1 into CC705. ST97 and ST705 have emerged as dominant lineages derived from bovine milk worldwide [31]. The other 3 isolates belonged to CC1, CC5 and CC8; these 3 CCs, thought to be of human origin, have recently begun to emerge within bovine *S. aureus* populations with ST1 and ST8 isolates identified as a causative agent in cases of bovine mastitis [30, 32].

All ovine and caprine strains were assigned to CC133 suggesting it to be a dominant population throughout Victoria, however a larger study is required to confirm this. These results, however, are consistent with a recent study on the global diversity of ovine *S. aureus* isolates which identified CC133 as a dominant clonal complex among Australian and European derived isolates and hypothesised the likeliness of a shared ancestor, as sheep were originally introduced into Australia from Europe [33]. Ovine and caprine *S. aureus* isolates from other countries irrespective of clonal complex have been shown to be close relatives, and it is hypothesised that this could be a result of the tendency for caprine and ovine dairy farming to be combined especially by small farms for conservation of forage. The eBURST analysis of ovine and caprine isolates predicted 3 groups with a single ancestral genotype CC133 (Table 2 and Fig. 4). These results suggest isolates are highly clonal but the limited data may bias this. Limited research has been done on clonal complexes of *S. aureus* amongst ovine and caprine dairy animals in Australia, however overseas work has shown similar findings to this study, with CC133 being a dominant lineage among small ruminants such as sheep and goats.

Antibiotics are used to treat and prevent mastitis in Australia, however their use is considered conservative so little effort has been made to monitor the emergence of resistance among ruminant *S. aureus* populations. Livestock associated methicillin resistant *S. aureus* (LA-MRSA) begun to emerge in bovine milk in Europe in the early 2000s; fortunately this has not been observed in Australia however it remains important to monitor in terms of public health as antibiotic resistance may transfer either via the food chain or through close contact with the cattle themselves [34]. No antibiotic resistance was found among isolates in this study, with the exception of a single isolate (Sa14-002) displaying resistance to penicillin. Of all antimicrobial resistance gene markers screened, this isolate also contained the only one found: the penicillin resistance gene *blaZ*. This isolate belonged to CC8, which has been associated with penicillin resistance before [35]. A previous study found penicillin resistance was exhibited by STs originally of non-bovine descent (including ST8), whilst the majority of STs originally of bovine descent (e.g. ST97 and ST705, both identified in this study) were susceptible [36]. Penicillin resistance has been previously identified among Australian *S. aureus* isolates collected from bovine mastitis cases between 1974 to 1979, although resistance decreased from 35 to 7.1 % respectively over this period; however penicillinase was produced by 62 % of isolates in 1979 [37]. The current information on antibiotic resistance among ovine and caprine isolates of animal or milk origin is lacking in Australia making it difficult to draw parallel conclusions to past or current levels of resistance. A lack of resistance towards cephems which are a group of broad spectrum third generation cephalosporins is not surprising as ceftiofur is the only third generation antibiotic registered for use in the Australian animal industry and is primarily utilised on individual animals rather than herds. It is registered to treat respiratory conditions in beef and dairy cattle; and footrot and mastitis in dairy cattle only. Its use in mastitis treatment is restricted to dry therapy only, making the likelihood of transfer to milk relatively low. A lack of resistance towards fluroquinolones and glycopeptides is also not surprising as these antibiotics are not used for livestock treatment in Australia [38]. Tetracyclines and macrolides on the other hand are widely utilised for livestock treatment in Australia however no resistance was observed towards these antibiotics in this study, and none of the associated genetic markers screened for were found. Overall the level of antimicrobial resistance was low which correlates with previous studies in Australia, which noted ampicillin or penicillin as the primary resistance phenotypes [37, 39]. To help control transmission of MRSA isolates into the dairy food chain, continued surveillance among ruminant herds and the screening of milk in Australia could s help prevent the incidence of MRSA in the future and monitor for overuse of certain antibiotics, thereby safeguarding the Australian dairy industry.

The presence of *S. aureus* in milk can be detrimental to human health if it produces enterotoxins, which when ingested can result in staphylococcal-induced food poisoning. Most milk for human consumption is pasteurised, however enterotoxins may survive this intervention, creating a risk to consumers [8]. Unpasteurised goat’s milk can legally be sold in several Australian States, and recent legislative changes in Australia allow the use of unpasteurised milk in cheese production highlighting the significance of enterotoxin surveillance in raw milk. The virulence profile of the bovine isolates was diverse with 2 bovine isolates harbouring 9 different classical SE genes. Two pathogenicity islands have been identified as common in animal associated *S. aureus* isolates; the bovine pathogenicity island (SaPIbov) and the enterotoxin gene cluster (*egc*) [40, 41]. SaPIbov carries *sec, sel* and *tsst-1* and the *egc* cluster carries *seg, sei, sem, sen, seo, yent1* and *yent2* [42, 43]. The *egc* cluster is among the most common combination of enterotoxins identified in overseas studies [40, 44, 45]. Bovine isolates Sa14-003 and Sa14-004 contained the *egc* cluster and a homolog of the SaPIbov cluster. The most frequent enterotoxins among the *S. aureus* isolates in this study was *sec* and *sel*. Other similar studies have also reported a high prevalence of *sec* among milk-associated isolates [30, 46–48].

The 2 ovine isolates contained *sec* and *sel*. Three of the caprine *S. aureus* isolates contained *sec* and *sel* (60 %). In previous studies it has been hypothesised that the *egc* locus is more prevalent in bovine *S. aureus* compared to caprine and ovine isolates suggesting an importance of the *egc* cluster in bovine mastitis, whilst the *sel* lacking variant of the SaPIbov has been observed in higher incidence in caprine and ovine isolates [40]. The *egc* island was only identified in bovine isolates in this study (29 %) and was absent in all caprine and ovine isolates, however *sel*-containing pathogenicity islands were identified at a higher frequency among caprine and ovine isolates (60 % and 100 %, respectively), compared to bovine isolates (17 %). Four isolates (2 bovine and 2 caprine) did not contain any of the enterotoxin genes screened.

Although a high diversity of SE genes was identified among isolates in this study, it is not indicative to the level of expression in raw milk. Raw milk provides an excellent medium for growth of staphylococci due to its high nutritional content. Although pasteurisation will generally inactivate most bacteria present, enterotoxins already produced may survive this treatment and can subsequently lead to outbreaks of illness [25, 49]. In Australia, staphylococcal food poisoning is not considered a notifiable disease; since onset is rapid and illness short-lived, the levels reported are likely under represented [50]. None of the isolates produced detectable SE (i.e. SEC or SED) at 12 °C (Fig. 5), suggesting such temperature abuse of milk does not represent a significant risk of enterotoxin production. Bovine isolate Sa14-003 produced SED enterotoxin at 16 °C; this toxin has been implicated in bovine mastitis [51]. As little as 17 ng of SE has been implicated in causing illness following consumption [25]. At 37 °C all isolates produced detectable levels of enterotoxins, highlighting the need to maintain cold chain conditions in foods, for example post milking of animals.

## Conclusions

In conclusion, overall genetic lineages of the Victorian isolates are akin to dominant and emerging livestock associated lineages seen worldwide and are not unique to Australia, however results suggest novel ST variants may also be emerging in Australia. Low levels of antibiotic resistance were identified among isolates, although it should be noted that a limited number of isolates were analyzed in this study. A range of enterotoxin genes were found, however results suggest maintaining the cold chain will minimise the risk of their production in milk. The incidence of mastitis has a negative impact on milk quality, and the liberalisation of international trade agreements with China, Japan and Korea will most likely increase the demand for Australian milk placing greater importance on milk quality in the future.

## Methods

### Bacterial isolates in this study

A total of 30 *S. aureus* isolates (1–4 per sample) were collected from milk and milk filter samples from 7 dairy farms (3 bovine: bov1, *n =* 8; bov2, *n =* 4; bov3, *n =* 4; 2 caprine: cap1, *n =* 5; cap 2, *n =* 4; cap3, *n =* 0; and 1 ovine: ov1, *n =* 5) from the South-East Australian State of Victoria [52]. Each farm was visited in December 2012 and March 2013. Milk samples were serially diluted and plated on Brilliance Staph 24 agar (Oxoid, Basingstoke, UK). Milk filter samples were homogenized for 2 min in a Lab-blender 400 model stomacher (Seward, Worthing, UK) prior to agar plating.

### PFGE subtyping

PFGE was carried out on all isolates of *S. aureus* isolated using a method previously described by McDougal et al. [23], with the following modifications: cell lysis was achieved by immersing the plugs in 3 ml of EC lysis buffer for 4 h at 37 °C; plugs were washed four times in TE buffer for 30 min at room temperature; plug slices were equilibrated for 30 to 45 min in a water-buffer mixture (180 μl/plug slice of sterile reagent grade water and 20 μl/ plug slice of 10× buffer), then digested with 3 μl (10 U) of *Sma*I restriction enzyme in 197 μl of 1× buffer (20 μl of 10× buffer in 177 μl of sterile reagent grade water) at 25 °C for 3 h. Electrophoresis was performed in a CHEF-DR III electrophoresis cell (Bio-Rad, Hercules, CA) with *Salmonella* Braenderup strain H9812 included as a standard ladder for gel comparison. Gels were stained in ethidium bromide (0.5 μg/ml water) for 30 min then destained for 1 h in distilled water and visualised by UV transilluminator. Using the BioNumerics version 6.5 (Applied Maths, Sint-Martens-Latem, Belgium) software, pair-wise similarities were calculated using the dice coefficient, with the optimisation and tolerance set to 1 %. From these similarities a UPGMA- generated (unweighted Pair Group Method with Arithmetic Mean) dendrogram was constructed.

### Whole genome sequencing

The genomic DNA of 13 *S. aureus* isolates (1 for each PFGE pulsotype identified) was sequenced using the illumina MiSeq platform. Genomic DNA extraction was carried out on an overnight culture grown in BHI broth using the DNeasy Blood and Tissue kit (QIAGEN, Hilden, Germany) according to the manufacturer’s instructions. The DNA preparations were resuspended in nuclease-free water and the quality and quantity of the genomic DNA preparation was assessed using the Qubit dsDNA HS assay kit (Thermo Fisher Scientific, Waltham, MA). The A260/A280 of each sample was read using spectrophotometric analysis to ensure an OD in the range of 1.8 to 2.0. DNA preparations were sent to the Ramaciotti Centre for Genomics (University of New South Wales, Sydney, Australia) for sequence ready genomic library preparation using the Nextera XT library prep kit (illumina, San Diego, CA). Subsequently 300 bp paired-end sequencing was performed using the illumina MiSeq platform. Raw reads were pre-processed to remove adapter sequences and low quality reads using the Trimmomatic version 0.22 software [53]. De novo assembly was performed using the SPAdes (Species Prediction and Diversity Estimation) genome assembler tool version 2.5.1 [4], based on an algorithm which employs multisized De bruijn graphs with K-mer values of ‘21, 33, 55, 77’ to construct the contiguous sequences (contigs) [54]. FASTA files generated were processed through the online gene annotator RAST (Rapid Annotation of microbial genomes using Subsystems Technology) to produce GENBANK files. The FASTA and GENBANK files were imported into the Geneious software platform version 7.1.6 (Biomatters, New Zealand) to create in an-house database of the contigs of each of the isolates for *in-silico* analysis.

### MLST subtyping of isolates

*In-silico* MLST analysis was performed on the assembled contigs to determine the sequence type (ST). The following seven housekeeping genes were used in the MLST scheme: carbamate kinase (*arcC*), shikimate dehydrogenase (*aroE*), glycerol kinase (*glp*), guanylate kinase (*gmk*), phosphate acetyltransferase (*pta*), triosephosphate isomerase (*tpi*), and acetyl coenzyme A acetyltransferase (*yqiL*). [55]. The *S. aureus* MLST alleles were extracted from the assemblies based on sequence similarity and entered into the MLST database (http://saureus.mlst.net last accessed 16th July 2015) to search against all MLST alleles for that gene. The closet matching MLST for each of the 7 housekeeping genes were selected. The allelic profile or sequence type (ST) was determined by entering the combination of all 7 MLST alleles into the database. Clonal complexes were assigned by eBURST analysis of all *S. aureus* strains in the MLST database using the conservative approach of 6 out of 7 matching alleles [24].

To compare the genetic relatedness of the 13 isolates from this study with isolates of dairy milk origin in Australia and globally, data was extracted from the MLST database. An in-house database of Australian dairy isolates was created by performing a query using the search terms ‘aureus’, ‘Australia’ and ‘MLST’. In order to make global comparisons a database was established for each animal group by conducting a query of the following search terms; ‘bovine milk’, ‘caprine milk’, ‘goat’s milk’, ‘sheep’s milk’, ‘ovine milk‘, ‘cow’, ‘milk’ or ‘milk tank’. A population snapshot for bovine and small ruminants (caprine and ovine) was diagrammed by eBURST to illustrate clusters of linked and unlinked STs and eBURST analysis, with the conservative approach of 6 out of 7 matching loci. In addition the origin of isolates belonging to the sequence types identified in this study were compared to the sequence types present in the MLST database in order to identify common sources of origin.

### In silico analysis of virulence gene markers

For comparative analysis, the known reference gene sequences for enterotoxin genes *sea* to s*ee*, *seg* to *ser*, *tst, yent1*, *yent2* as well as *nuc* were retrieved from the NCBI (National Center for Biotechnology Information) database and imported to the Geneious software. A BLAST (Basic Local Alignment Search Tools) search was conducted on the in-house database to determine the presence or absence of the gene markers among isolates in this study. A similar approach was used to identify the presence of antimicrobial gene markers among the 13 isolates. Results of the enterotoxin BLAST analysis was confirmed using previously published primers targeting enterotoxin genes [56–58].

### Antibiotic sensitivity testing

The evaluation of antimicrobial susceptibility of all 13 isolates to a panel of 7 antibiotics (ceftriaxone, ciprofloxacin, erythromycin, oxacillin, penicillin, tetracycline and vancomycin) were assessed using M.I.C. Evaluators™ (Oxoid), as per manufacturer’s instruction. In brief, isolates were removed from the microbank strorage system at −80 °C and plated onto tryptone soy agar (TSA, Oxoid) overnight at 37 °C. For each isolate an inoculum was prepared in modified Mueller Hinton broth (cation adjusted Mueller Hinton broth with TES; Trek Diagnostics systems, UK) and adjusted to 0.5 McFarland. The adjusted bacterial suspensions were inoculated onto Mueller-Hinton agar plates (Oxoid) to form an even lawn; excluding oxacillin which was inoculated onto Mueller-Hinton agar supplemented with 2 % sodium chloride. The M.I.C. Evaluator™ strips were added to the plates according to the manufacturers’ instructions and were incubated for 20 to 24 h at 35 °C. For erythromycin, oxacillin, penicillin G, tetracycline and vancomycin, 0.015–256 μg/ml strips were used; for ceftriaxone and ciprofloxacin, 0.002–32 μg/ml strips were used. The minimum inhibitory concentration (MIC) for each antibiotic was determined by examining the zone of inhibition following the manufacturer’s instructions, for all 13 strains. Antibiotic sensitivities (‘sensitive’, ‘intermediate’, or ‘resistant’) were assessed according to the Performance Standards for Antimicrobial susceptibility testing, twenty-fourth informational supplement, (January 2014) and European Committee on Antimicrobial Susceptibility Testing 2014 (http://www.eucast.org/clinical_breakpoints) [59]. Antimicrobial gene markers screened for among isolates in this study comprised *mecA*, *mecC*, *blaZ*, *vanA*, *vanB*, *vanC, tetK*, *tetM*, *ermA*, *ermC* as well as the *aacA*-*aphD* aminoglycoside resistance determinant, using gene BLAST analysis and previously described screening primers [60–62].

### Enterotoxin detection in milk

Five *S. aureus* isolates (2 bovine, 2 caprine, 1 ovine) were selected for quantification of *Staphylococcus* enterotoxin production in 10 % reconstituted milk (RSM) at 12 °C, 16 °C and 37 °C. The *S. aureus* strains were inoculated into 10 ml RSM (Oxoid) at a concentration of 4 log CFU/ml. Spiked milk samples for each strain were incubated at 12 °C, 16 °C and 37 °C for 72 h, 48 h and 18 h respectively. Post incubation the level of *Staphylococcus* enterotoxin A, B, C, D and E was determined by sandwich enzyme immunoassay (Ridascreen set A, B, C, D, E, R-biopharm AG, Darmstadt, Germany) following the manufacturer’s instructions. In brief, 100 μl of spiked milk sample was added to row A to G of a microtiter strip and incubated at 35 °C for 1 h. Post incubation the wells were washed five times in 300 μl of wash buffer. Then 100 μl of conjugate 1 was added and the wells were incubated for 1 h at 35 °C. The wells were then washed another five times in 300 μl of wash buffer and 100 μl of conjugate 2 was added. The wells were incubated for 30 min at 35 °C followed by a final wash step carried out five times with 300 μl of wash buffer. For colour development 100 μl of substrate/chromogen was added to the wells and incubated for 15 min at 35 °C. To stop the reaction 100 μl of stop solution was added and the absorbance was read immediately at 450/630 nm.
